# Effect of the ratio of axial length to keratometry on SRK/T intraocular lens power calculations for eyes with long axial lengths

**DOI:** 10.1038/s41598-019-56116-4

**Published:** 2019-12-20

**Authors:** Yosai Mori, Keiichiro Minami, Shota Tokuda, Jinhee Lee, Kazunori Miyata

**Affiliations:** Miyata Eye Hospital, Miyakonojo, Miyazaki Japan

**Keywords:** Outcomes research, Risk factors

## Abstract

This retrospective study explored the effect of the ratio of axial length (AL) to average keratometry (K) on intraocular lens power calculation in long eyes. The clinical records of eyes that had an AL of 26.0 mm or longer, and underwent cataract surgery with intraocular lens implantations, were reviewed. This study was approved by the institutional review board of Miyata Eye Hospital. Preoperative biometry data were obtained using optical low-coherence reflectometry. Prediction errors in the use of the SRK/T formulas were obtained from manifest refraction spherical equivalents one month postoperatively. Significant factors inducing prediction errors were examined using stepwise multiple regression analysis with descriptive factors of AL, K value, and their ratio (AL/K). Clinical records related to 49 long eyes of 49 patients, and 93 eyes of 93 patients with normal AL, were evaluated. Stepwise multiple regression analysis revealed that the AL/K was a significant factor increasing the prediction errors (P = 0.0003). With the regression equation, 98% of prediction errors with the use of the SRK/T formula were within ±1.00 D of differences. For our sample of 49 long eyes, the ratio of AL to K was a significant factor inducing hyperopic prediction errors with the use of SRK/T for long eyes.

## Introduction

The third and fourth generation intraocular lens (IOL) calculations, such as the SRK/T and Haigis formulas are comprehensively used^1,2^, and refraction errors can be corrected within 0.50 diopter (D) from the intended refractions in more than 70% of the cases^3,4^. However, the refractive errors for long eyes with an axial length (AL) over 26.0 mm is still insufficient^5–8^. Proportion of myopes with relatively long AL have been increasing in Asia region^9^. Inaccurate AL measurement for long eyes is considered one of the factors inducing the refractive error, and adjustments in the measured AL have been proposed^6,7^. On the other hand, the use of new-generation formulas, such as the Barrett Universal II (Barret UII) formula^8^ minimizes such refractive errors without AL adjustment^7,10^, indicating that the accuracy in AL measurement is not critical. The reason why hyperopic refractive errors occur for long eyes is not established, while it would be important to identify the critical factors for developing future IOL power calculations.

The cornea of a long eye is flatter than those of normal eyes, and the average keratometry (K) value in diopter decreases with AL^11–13^. A recent studies by Reitblat *et al*. and Melles *et al*. revealed that the flat cornea (K value less than 42.0 D) induced hyperopic refractive errors when the SRK/T formula was applied^10,14^. The SRK/T formula calculates an effective lens position (ELP) based on corneal height and offset, where the corneal height is geometrically obtained from a curvature of the corneal sphere and estimation of the corneal width^1^. Consequently, the ELP increases with AL. Whereas, an epidemiological assessment of 1184 eyes shows that the anterior chamber depth (ACD) does not alter with AL for long and extremely long eyes^15^. The previous findings indicate that ELP would be overestimated in long eyes^5,10^. Melles *et al*. demonstrate that eyes with a longer AL or lower K values induce hyperopic errors in the use of SRK/T formula^10^. Hence, it was speculated that a particular relation between the AL and K values would be a critical factor of the hyperopic error. However, the effect of this relationship, to our knowledge, has not been investigated. This study aimed to explore the effect of the relation between the AL and K values on intraocular lens power calculations in long eyes.

## Results

**Figure 1:**
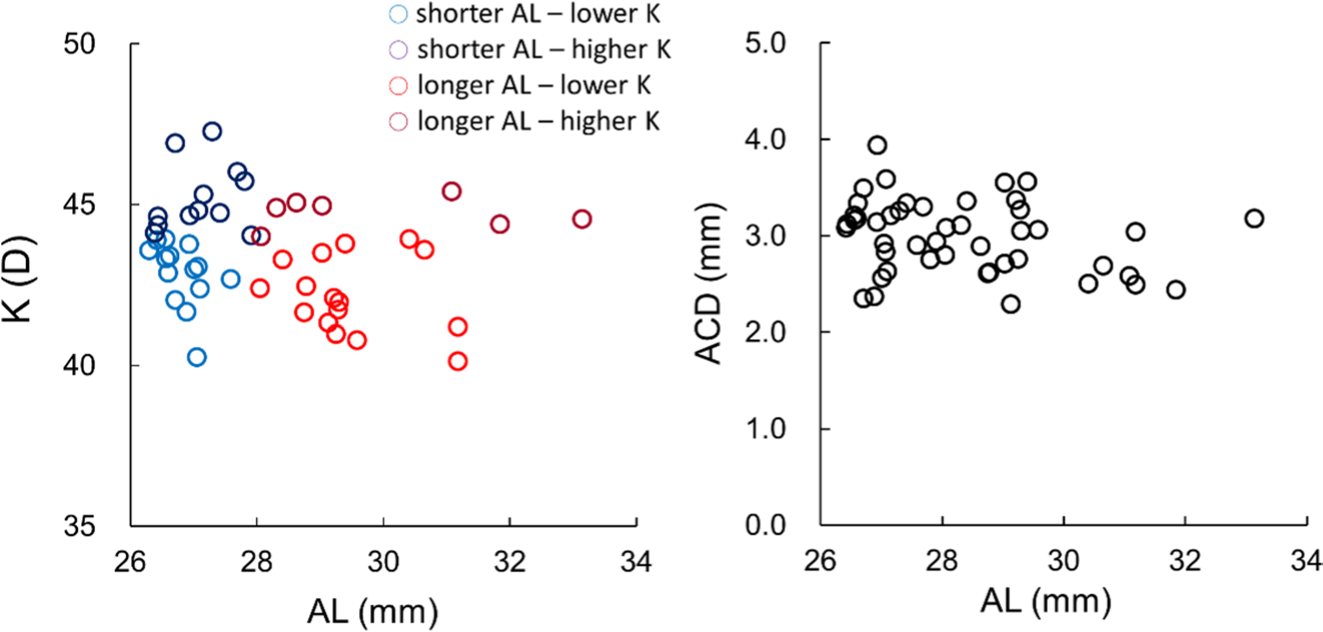
Relationship of average keratometry (K) value (left) and anterior chamber depth (ACD, right) with axial length (AL). Neither the ACD nor the K was associated with the AL (P = 0.084, and 0.19, respectively). In the left plot, the data were divided into 4 quadrants with borders of the AL of 28 mm and the K of 44 D, so that there were 4 regions of a short AL and lower K, shorter AL and higher K, longer AL and lower K, and longer AL and higher K.

Clinical records of 49 eyes of 49 patients with an AL of 26.0 mm or longer were evaluated in the study. The mean age of the included population was 60.3 years (standard deviation [SD]: 6.7 years), and the implanted IOLs consisted of SN60WF (Alcon, Fort Worth, USA) in seven eyes, ZCB00/V (Johnson & Johnson Surgical Vision, Santa Ana, USA) in 35 eyes, and VA60BB/YA60BBR (HOYA, Tokyo, Japan) in seven eyes. The normal eyes consisted of 93 eyes of 93 patients. The mean age of patients was 72.2 years (SD: 6.8 years), and the implanted IOLs consisted of SN60WF in 61 eyes, ZCB00/V in 17 eyes, and YA60BBR in 15 eyes. Table 1 shows the demographic data of the subjects. The ACD and IOL power of the long eyes were significantly lesser than those of the control eyes (P < 0.033, t-test). The corneal astigmatism was significant higher in the longer eyes (P = 0.01), but the difference was 0.3 D. Figure 1 shows the changes in the preoperative K-value and ACD with the AL in the long eyes; no significant change was observed (P = 0.19 and 0.084, linear regression analysis, respectively). In the left plot, the data were divided into 4 quadrants with borders of AL of 28 mm and K of 44 D, so that there were 4 regions of a short AL and lower K, shorter AL and higher K, longer AL and lower K, and longer AL and higher K. With the sample size (N = 49) and a significance level of 0.05, the detection power in multiple regression analysis of a factor was anticipated to be 0.94 and 0.65 when R^2^ was 0.2 and 0.1, respectively.

**Table 1:** Demographic data of long and control eyes.

No. of eyes	Long eyes Mean ± SD (Range)	Normal eyes Mean ± SD (Range)	*P*, *t*-test
	49	93	
Axial length (mm)	28.21 ± 1.66 (26.30 to 33.14)	24.03 ± 0.57 (22.57 to 25.48)	<0.001
Mean keratometry (D)	43.5 ± 1.6 (40.1 to 47.3)	43.8 ± 1.3 (39.8 to 46.3)	0.31
Corneal astigmatism (D)	1.1 ± 0.6 (0.0 to 3.3)	0.8 ± 0.6 (0.0 to 3.0)	0.01
ACD (mm)	2.98 ± 0.61 (1.75 to 3.94)	3.13 ± 0.38 (2.31 to 3.97)	0.033
IOL power (D)	9.7 ± 4.3 (−3.0 to 19.0)	19.5 ± 1.8 (14.0 to 23.0)	<0.001

SD: standard deviation; D: diopter; ACD: anterior chamber depth; IOL: intraocular lens.

Table 2 shows the mean numerical prediction error (ME), median of absolute prediction error (MedAE), and eyes within ±0.50 D and ±1.00 D prediction errors with the use of SRK/T and Barret UII for the long and normal eyes. The ME in the long eyes were significantly higher than in the normal eyes (P < 0.001). Comparing with the Barrett UII, there was no significant difference in the ME and MedAE in the long eyes (P > 0.02). The prediction error with the SRK/T changed with the AL (P < 0.001, R^2^ = 0.24, linear regression analysis), while such an alternation was not found with the use of Barrett UII (P = 0.85). The prediction errors of 2 kinds of IOL shapes, biconvex (SN60WF and ZCB00V) and meniscus (VA60BB/YA60BBR) IOLs, were also compared in long eyes with the use of SRK/T. Table 3 shows the results: The ME and MedAE were significantly larger in the use of the meniscus IOLs (P < 0.04, Man-Whitney test).

**Table 2:** Mean numerical prediction error (ME), median of absolute prediction error (MedAE), and eyes within ±0.50 D and ±1.00 D of prediction errors with the use of SRK/T and Barrett Universal II formulas for long and normal axial length eyes.

Formula	SRK/T			Barrett Universal II			
	Long eyes	Normal eyes	P-value^(a)^	Long eyes	P-value^(b)^	Normal eyes	P-value^(a)^
ME, D (range)	0.39 ± 0.47 (−0.50 to 1.82)	0.00 ± 0.47 (−1.16 to 1.45)	<0.001^(c)^	0.33 ± 0.41 (−0.49 to 1.33)	0.20^(d)^	0.18 ± 0.42 (−0.82 to 1.25)	0.048^(c)^
MedAE, D (range)	0.46 (0.00 to 1.82)	0.31 (0.00 to 1.45)	0.18^(e)^	0.38 (0.00 to 1.33)	0.40^(f)^	0.28 (0.00 to 1.25)	0.094^(e)^
Within ±0.50 D error, %	57.1%	73.1%		67.3%		76.9%	
Within ±1.00 D error, %	91.8%	95.7%		95.9%		96.2%	

Mean ± standard deviation.

D: diopter; ^(a)^between long and normal eyes; ^(b)^between SRK/T and Barrett Universal II for long eyes; ^(c)^t-test; ^(d)^paired t-test; ^(e)^Mann-Whitney U test; ^(f)^Wilcoxon signed rank test.

**Table 3:** Mean numerical prediction error (ME), median of absolute prediction error (MedAE), and eyes within ±0.50 D and ±1.00 D of prediction errors with the use of SRK/T for biconvex and meniscus intraocular lenses (IOLs) in long eyes.

IOL	SRK/T in long eyes		
	Biconvex (N = 42)	Meniscus (N = 7)	P-value^(a)^
ME, D (range)	0.29 ± 0.37 (−0.50 to 0.93)	0.93 ± 0.69 (−0.01 to 1.75)	0.015^(b)^
MedAE, D (range)	0.43 (0.00 to 0.93)	1.07 (0.01 to 1.75)	0.040^(b)^
Eyes within ± 0.50 D error, %	61.9%	28.6%	
Eyes within ± 1.00 D error, %	100%	42.9%	

Mean ± standard deviation.

D: diopter; ^(a)^between biconvex and meniscus IOLs; ^(b)^Mann-Whitney U test.

**Figure 2:**
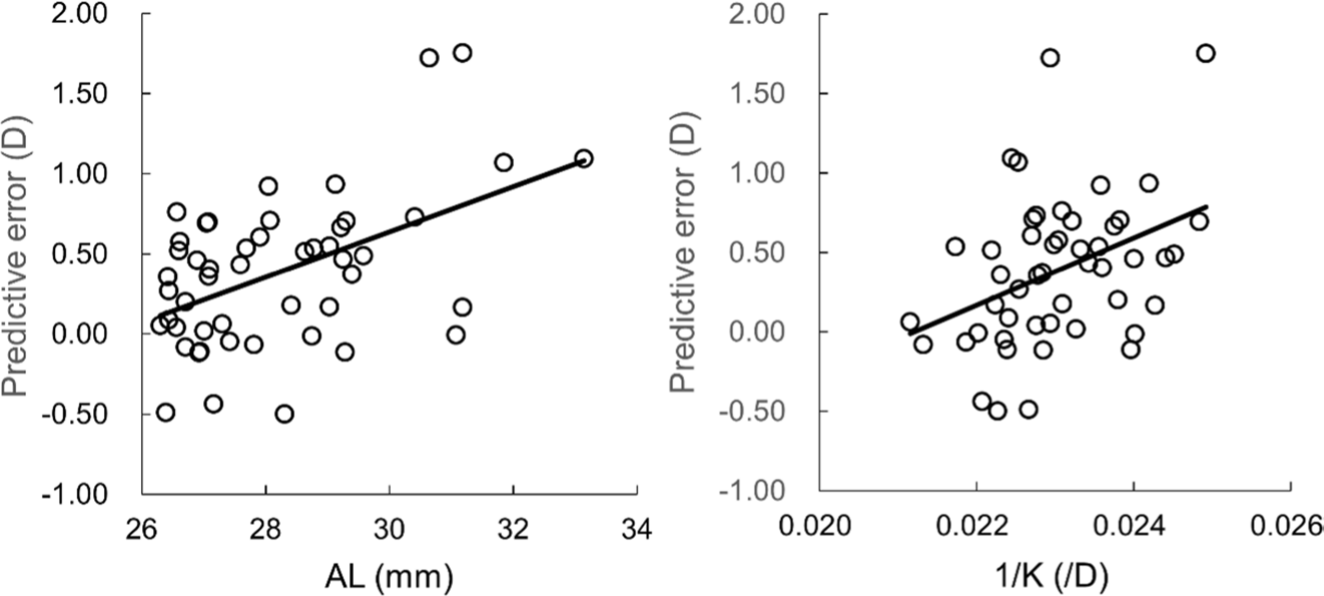
Relationships between the prediction errors and the AL and 1/K values. There were significant correlations with the AL and 1/K (P < 0.006, R^2^ < 0.25).

**Figure 3:**
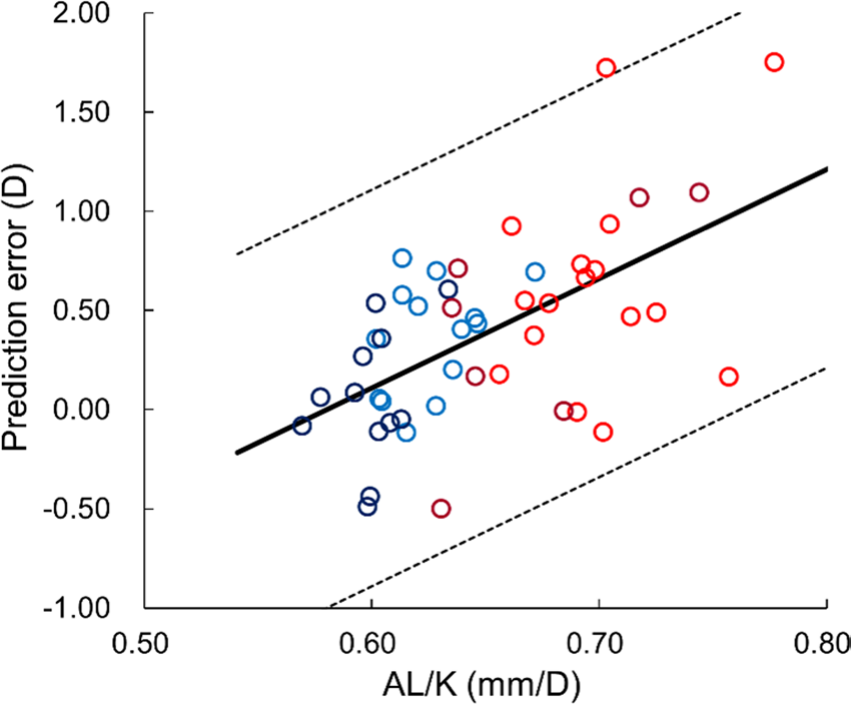
Relationship of the ratio of axial length (AL) to the average keratometry (K) values, (AL/K), with the prediction errors in the use of SRK/T. The solid line indicates the regression equation with a significant factor of AL/K (P < 0.001, R^2^ = 0.33), and dotted lines indicate ±1.00 D differences from the regression equation. Colors of plotted points corresponded with the 4 regions defined in the left plot of Fig. 1.

The ratio of the AL to the K values (AL/K) were in a range of 0.57 to 0.78 (mean: 0.65). Figure 2 shows the relationships between the prediction errors and the AL and 1/K values. The regression analysis showed that the correlations with the AL and 1/K were significant (P < 0.006, R^2^ < 0.25). The stepwise multiple regression analysis for the use of the SRK/T resulted that the AL/K was a significant factor (P < 0.001, R^2^ = 0.33). As shown in Fig. 3, the regression equation was obtained as prediction error = 5.5093 × AL/K − 3.1964. There were 79.6% and 98.0% of eyes within ±0.50 D and ±1.00 D differences from the regression equation, respectively. The ratio within ±0.50 D difference was significantly higher than with the use of SRK/T only (P = 0.029, Fisher’s exact test). The stepwise multiple regression analysis for the Barrett UII resulted in no significant factor.

## Discussion

In long eyes, prediction errors increased with the AL with the use of SRK/T formula, although such an increase was not found with the Barrett UII formula. The stepwise multiple regression analysis revealed the contribution of AL/K in the use of the SRK/T formula. Previous studies have compared the prediction errors in long eyes using various calculations^5–7,10^, and the current results coincided with the results of these studies. However, in our knowledge, the current study firstly evaluated the effect of the AL/K has not been examined.

The prediction error with the use of the SRK/T formula was associated with the AL. As one of causes, inaccurate AL measurement in long eyes has been addressed, and adjustment of AL values improved the accuracy of SRK/T calculation^6,7^. Comparisons by Abulafia A *et al*. showed that there were 65.8–66.7% and 96.7–100% of eyes within ±0.50 D and ±1.00 D of prediction errors^7^, which was comparable to the results in Fig. 2. Preferable accuracy in the use of the Barrett UII without AL adjustment suggested that refractive errors in log eyes would be inherently related with the SRK/T^7,10^. The SRK/T assumes a gradual increase in ELP with AL, while the preoperative ACD in the current and previous^15^ studies did not vary with the AL. The current analysis results indicated that the deviation in the AL/K would be inherent from the use of SRK/T for long eyes. Further assessment with sufficient sample size is necessary for confirmation.

The Barret UII provided superior outcomes in the long eyes. The formula models the entire eye with two spheres: the corneal and global spheres^8^. The radius of the corneal sphere is calculated from K value, while the radius of the global sphere is obtained with AL and K values. Hence, the relationship between AL and K value is not assumed. Previous studies indicate that the use of the multiple spheres modeling enables more accurate power calculation beyond the use of SRK/T formula^7,10,16^.

There were limitations in the current study. Owing to the retrospective nature of the cases, there were variations in the implanted IOL models. In practice, it was difficult to restrict IOL models because the IOL powers could not cover all long eyes with a single model. The one-piece IOLs of SN60WF and ZCB00/V were available in the power of 6.0 D or higher, whereas the meniscus-shaped VA60BB/YA60BBR was used for IOL powers below 6.0 D. Differences of multiple models were compensated for by optimizing the A-constants. However, a prospective design using one or two models of IOL would be desirable for further assessments. Next, the sample size (N = 49) was not sufficient. In the multiple regression analysis of SRK/T values, the resultant R^2^ was resulted in 0.33, which corresponded to the anticipated detection power of 0.98. Additionally, the values of R^2^ was comparable to the previous similar analysis (R^2^ = 0.23–0.36)^17,18^. Hence, the effect of the AL/K would be acceptable for demonstrating the effect of AL/K. A larger sample size is desired for validating the effect of the ratio. The other limitation would be preoperative biometry. Biometry data were measured with an optical low-coherence reflectometry utilizing a time-domain interferometry. The use of Fourier-domain interferometry, such as swept-source optical coherence tomography^19,20^ allows higher repeatability in biometry measurements. Lastly, the current study was aimed to examine the effect of AL/K only, so the relation obtained could not be used clinically. There are other factors inducing the refractive errors such as insufficient accuracy in biometry and postoperative examination, tolerance of IOL power (approximately within 0.4 D)^21^, asymmetry in convex shapes, and corneal asphericity^17,18^. So, more compensation is necessary to improve most of long eyes. However, it is anticipated that the revealed results would be useful for further understandings.

In conclusion, it was demonstrated that the change in the ratio of the AL to the K values was a factor inducing hyperopic postoperative refractive errors in the use of SRK/T formula for long eyes.

## Methods

### Subjects

This retrospective case series was approved by the Institutional Review Board of Miyata Eye Hospital, Miyazaki, Japan, and followed the tenets of the Declaration of Helsinki. Informed consent for use of the clinical data was obtained from all patients. Clinical records of patients who had an AL of 26.0 mm or longer and underwent cataract surgery with monofocal IOL implantation were reviewed. Inclusion criteria included IOL implantation within the capsular bag and postoperative corrected visual acuity (BCVA) of 0.2 logMAR or better. The inclusion criterion of BCVA was determined for obtaining prediction errors accurately. Eyes with intra- and post-operative complications due to the cataract surgery or with any other ocular pathology influencing the BCVA were excluded. Fundoscopy or optical coherence tomography were performed preoperatively for examining staphyloma, foveal atrophy and degeneration, and other abnormality of the retina. In bilateral cases, the first eye was used for analysis. Patients with normal ALs between 23.5 and 26.0 mm were also included as control.

### Examinations

Preoperative AL, K value, ACD, corneal diameter, and lens thickness were measured using optical low-coherence reflectometry (Lenstar^®^ LS-900 with a software En Suite^®^ i8.2.1.0, Haag-Steit, Bern, Switzerland). The low-coherence reflectometer with a super-luminescent diode (wavelength: 820 nm) was used to measure the AL, ACD, and lens thickness. The K values were obtained from images of the cornea, on which 32 points on 2 rings of 1.65 and 2.30 mm diameter were projected, and calculated using a refractive index of the cornea of 1.3375. Powers of implanted IOLs were determined using the SRK/T formula together with A-constants available in the User Group for Laser Interference Biometry (http://ocusoft.de/ulib/c1.htm). Manifest refraction spherical equivalent was measured one month postoperatively.

### Statistical analysis

Predicted postoperative refractions were obtained after optimization. A-constants for IOLs used were optimized using outcomes of the normal eyes, and the optimized A-constants were used for the long eyes for compensating differences in the IOL model. Difference between the predicted postoperative refractions and manifest refractions were calculated as prediction errors. The ME, MedAE, and percentages of eyes within ±0.50 D and ±1.00 D, were calculated for long and normal eyes. Differences in ME were examined using t-test, while those in MedAE were examined using nonparametric Mann-Whitney or Wilcoxon tests. Relationship between the prediction errors and AL were evaluated using linear regression analysis.

The effect of the ratio of AL to K vales (AL/K) was examined in long eyes. Association of AL/K on the prediction errors was evaluated by a stepwise multiple regression analysis^17^. For the objective factor of prediction error, the descriptive factors were chosen to be AL, K value, and AL/K. When AL/K was selected as a significant factor, difference of the prediction errors from the resultant regression equation were calculated. The above analysis was also performed with the use of Barrett UII was used in the long eyes. P < 0.05 was considered significant.

## Data Availability

The datasets generated during and/or analyzed during the current study are available from the corresponding author on reasonable request.
